# Evaluating and Validating Emotion Elicitation Using English and Arabic Movie Clips on a Saudi Sample

**DOI:** 10.3390/s19102218

**Published:** 2019-05-14

**Authors:** Sharifa Alghowinem, Roland Goecke, Michael Wagner, Areej Alwabil

**Affiliations:** 1 College of Computer and Information Science, Prince Sultan University, Riyadh 11586, Saudi Arabia; 2 Research School of Computer Science, Australian National University, Canberra 2600, Australia; michael.wagner@canberra.edu.au; 3 School of Information Technology & Systems, University of Canberra, Canberra 2600, Australia; roland.goecke@ieee.org; 4 Center for Complex Engineering Systems, King Abdulaziz City for Science and Technology, Riyadh 11586, Saudi Arabia; areej@mit.edu

**Keywords:** affective computing, cross-culture, emotion elicitation, emotion recognition, physiological responses, emotion stimuli

## Abstract

With the advancement of technology in both hardware and software, estimating human affective states has become possible. Currently, movie clips are used as they are a widely-accepted method of eliciting emotions in a replicable way. However, cultural differences might influence the effectiveness of some video clips to elicit the target emotions. In this paper, we describe several sensors and techniques to measure, validate and investigate the relationship between cultural acceptance and eliciting universal expressions of affect using movie clips. For emotion elicitation, a standardised list of English language clips, as well as an initial set of Arabic video clips are used for comparison. For validation, bio-signal devices to measure physiological and behavioural responses associated with emotional stimuli are used. Physiological and behavioural responses are measured from 29 subjects of Arabic background while watching the selected clips. For the six emotions’ classification, a multiclass SVM (six-class) classifier using the physiological and behavioural measures as input results in a higher recognition rate for elicited emotions from Arabic video clips (avg. 60%) compared to the English video clips (avg. 52%). These results might reflect that using video clips from the subjects’ culture is more likely to elicit the target emotions. Besides measuring the physiological and behavioural responses, an online survey was carried out to evaluate the effectiveness of the selected video clips in eliciting the target emotions. The online survey, having on average 220 respondents for each clip, supported the findings.

## 1. Introduction

It has been long argued whether emotions are universal or influenced by culture [1,2,3,4]. With the advancement of technology, validating this argument can be complemented using physiological sensors and automatic recognition of responses. Such validation is important not only to gain better insight into human psychology, neurobiology and sociology, but also to boost the development of emotionally-intelligent systems that react based on the users’ affective state [5]. With the fast progression of current sensor technology, it is possible to expand, advance and validate emotionally-intelligent systems. For example, the use of bio-signal sensors to measure physiological and behavioural responses adds another dimension to validate objectively the effectiveness of such an intelligent system. These emotionally-intelligent systems could improve education (e.g., adjusting the teaching style based on the emotional state of a student [6]), marketing (e.g., advertisement personalisation [7]), surveillance (e.g., detecting suspicious behaviour [8]), psychology (e.g., diagnosing mental disorders [9]), and many other disciplines.

To commence such development, emotional responses should be elicited using stimuli in order to be measured. Such stimulus should be standardised to elicit single targeted emotion among different subjects [10]. Therefore, several methods have been investigated and validated for their effectiveness in inducing emotions [11]. Amongst the many different methods to elicit emotions, video clips are widely used for this task due to their advantages compared to other techniques [12,13]. Since video can often portray much more than the written word in reflecting on scenes and events in everyday life, they can elicit strong subjective and physiological changes. At the same time, video clips can induce mixed emotions with varied arousal, which therefore should be selected carefully to have a valid and reliable emotional responses [10]. The work in [10] listed several criteria for video clips selection including clip duration and intensity of elicited emotion, etc. Moreover, the onset, peak and offset of inducing emotion using a clip need to be correlated with the time line of of the clip. For example, a list of validated (English language) clips has been standardised and made available for such studies [10]. However, this list and most other collected stimuli lists have been validated without accounting for cultural influence on the elicited emotion. Therefore, with the cultural differences, it is possible that some of the validated clips, even when dubbed, will not elicit similar responses in participants from different cultures. That is, some of the video clips may have cultural value conflicts, which might make them culturally unacceptable by another community and, therefore, lose the effect in inducing the target emotion.

Given the unique and conservative Arabic culture, there is a need to evaluate and validate a set of emotion-eliciting video clips that are suitable for Arabic speakers to be used in affect studies on an Arab population. In particular, this paper evaluates and validates the relationship between cultural acceptance of emotional stimuli with their emotional response, by performing a comparative analysis of stimulus video clips in one culture. In an attempt to answer this paper’s research question, the following objectives are formulated:

- To evaluate the suitability of the validated English set of emotion elicitation clips from [10] in eliciting the target emotions in the Arab sample (Saudis in particular).
- To compare the evaluation results from the English set with an initial selection of the Arabic set of clips that is designed to elicit the same set of emotions as the English clips.
- To validate the evaluation results by analysing and classifying emotions based on the bio-signal and behavioural responses acquired during recording sessions in a laboratory environment.

It is worth noting that this study does not aim to collect standardised Arabic video clips that could be used for emotion elicitation. Rather, it investigates weather the already standardised clip could be utilised for this purpose in Arab culture. A more advanced framework for standardising emotion stimuli was proposed in [14,15]. Moreover, we hypothesise that English stimuli would have less effect in inducing emotions compared to an Arabic stimuli both in subjective (self-report) and objective (physiological responses) measures, even when following cultural constraints.

## 2. Background

### 2.1. Emotion and Emotion Stimuli

Precisely defining the concept “emotion” is one of the issues faced in the affective computing field. Psychologically, affect is a general concept, which covers not only emotions, but also mood, attitudes, etc. [16]. Emotions are defined as states of feeling that are short-lived, more or less intense and related to a recognisable object [17], yet classifying one affective state from the other is highly controversial. Emotion representations that have been studied in the literature could be divided into simple (positive vs. negative), discrete (specified set of emotions) and dimensional representations. Ekman, for example, studied six basic emotions (amusement, sadness, surprise, fear, anger and disgust) that are recognised universally [18]. However, as emotions could be more complex and blended, several alternative ways have been used to define emotions by using multiple dimensions or scales. A common way to describe emotion is Russell’s model [19], which uses a two-dimensional plane, where valence and arousal form the two axes [20]. In addition, a three-dimensional emotion model was proposed by [21] as: “pleasurable versus unpleasurable”, “arousing or subduing” and “strain or relaxation”. For simplicity, this work uses the basic six emotions suggested by Ekman, as they also have been proven to be universally recognised across cultures [22]. Nevertheless, we believe that the method presented in this research could be used for continuous space emotion representation and that using only six emotions is not a limiting factor for such studies.

Several standardised techniques to elicit discrete emotions have been used (e.g., imagination, music, texts, clips) [23,24]. Video clips are widely employed for their standardisation ability and their results’ replicability in inducing emotions [10,24]. Several studies collected and validated film clips that elicited different emotions [10,25,26,27,28,29]. Criteria to select the clips were the (1) length, where it is preferable to be relatively short, (2) understandability, where no additional explanation is required for understanding the clip content, and (3) discreteness, where the clip is likely to elicit one discrete emotion [10]. The range of clips’ length collected by these studies was between 0:09 min and 7:58 min, with an average length of 2:28 min. The earliest study by [25] collected 12 emotions’ elicitation Western film clips to elicit six emotions and was validated on 60 French-speaking Belgian students. Afterwards, the work in [10] collected over 250 Western films for evaluation, which were short-listed to a 78-clip list. They showed the short-listed clips to 494 English-speaking subjects for self-rating, where a smaller list of 16 films that elicited eight targeted emotions was standardised. However, these lists could be considered outdated. Recently, the work in [28] validated a list of 70 French-speaking video clips collected from different cultural backgrounds (French, Italian, British, and American) on 364 subjects. Another recent study validated a Spanish-dubbed set of clips that elicited seven emotions (including neutral) on 127 subjects [30]. The latest study by [29] validated 199 English YouTube film clips that elicited positive, negative and mixed emotions on 411 subjects. However, the mentioned standardised lists were validated for a specific culture. Cultural differences from both the selected clips and subjects were not accounted for, which could play a critical role in emotion trigger for the audience, as discussed in the following section.

### 2.2. Cross-Cultural Studies on Emotion Elicitation

The cultural background could have an influence on emotions felt and their triggers, where expression and interpretation of an emotion drastically differs between cultures [3,22]. Differences between cultures lie in the cultural influence on emotional responses to emotional stimuli [31]. The previous study supported the conclusion with an example of differences between the reactions of North and South Americans to the same emotion-eliciting stimuli [31]. Therefore, the same set of elicitation clips could produce different results for different cultures. Cross-cultural studies have been conducted to show the universality of emotion interpretation and expression [1,2,4,32]. Regardless of the partial differences in emotion interpretation and expression, an agreement has been reached over a universal set of emotions, which are widely used in the automatic recognition of emotions and affective computing studies. In [26], 45 German subjects participated in group film-viewing sessions, then were asked to rate their experienced emotion by each clip. Although only four video clips have been used from the list of [10], those four clips have been rated highly for their targeted emotions, which suggests universal elicitation of affect. In [33], clips [10] were used to elicit seven emotions on 29 undergraduate students. The used clips induced the target emotions except for the anger clips. Interestingly, the work in [22] showed the English clips from [10] to a Japanese sample of 31 participants to study the effectiveness of these clips in inducing the target emotions. The study found that some of the clips also resulted in the elicitation of non-target emotions, which appeared to be specific to the Japanese culture [22]. This finding emphasises the need to validate emotion stimuli for the suitability of intended subjects and their cultural background.

Investigations of the influence of religion on culture and social behaviour have been studied and well documented [34,35,36,37,38]. Arab culture emphasises the respect for all religions, honouring and obeying parents and following and complying with Islamic religious rules [39]. The work in [35] studied advertisement in Saudi Arabia in particular, where the study showed a detail guidelines for compliance to religion and regulation to ensure cultural acceptance and therefore the potential success of the advertisement campaign. Similarly, clip selection should be aligned with eligibility criteria, best practices and local guidelines [35]. For example, religious socio-cultural norms prohibit any content that presents (1) strict taboos such as alcohol, gambling and sexual references and (2) conflict with Muslim obligations such as disrespectful behaviour to parents and elders, even in a humorous context [35].

### 2.3. Automatic Recognition of Emotion

Many researchers have studied several potential signals to detect emotions automatically and objectively [40]. The most common approach is to use vocal and facial expressions. Detecting emotion from speech has been a focus for several decades in the affective computing field, with diverse applications [41]. For example, the work in [42] aimed at building smart security voice systems where they studied the emotional impact on human speech, especially when stressed. However, these signals might not be sufficient if the subjects want to conceal their emotions. Therefore, several studies also investigated physiological signals such as: heart rate, skin conductivity, brain waves, etc., for the task of detecting and understanding emotions.

Facial expression of emotion is the most obvious and common way to express and interpret emotions. Most automated facial emotion recognition systems use measurements from the muscles’ movement, which were identified by [43]. In their Facial Action Coding System (FACS), sets of Action Units (AUs) identified independent motions of the face, from which an expression could be recognised. By observing which AUs had been accrued, a specific expression would be classified and linked to the six basic emotions. However, manually coding FACS is time consuming and requires expert knowledge, which is considered to be an obstacle to the study of affect [44]. Fortunately, computer vision techniques can overcome this obstacle [44]. In addition, simple behaviours such as head movements reflect cues about mood, emotions, personality or cognitive processing [45]. That is, the pattern of the head node, the speed and frequency of head movements and the direction and the orientation of the head pose could be linked to different emotions. For example, head shaking has been associated with surprise and automatically and objectively head with sadness emotion [46].

While facial expressions could be concealed, eye activity and pupil size are mostly involuntary. Several studies have explored detecting a person’s state using eye tracking techniques, such as: driver fatigue [47], cognitive load [48,49] and eye responses to emotional stimuli [50,51,52,53]. Eye activities such as pupil dilation, blinking and fixation could provide cues about the emotional dimension such as arousal or valence, which are related to various emotions. Therefore, it has been suggested that eye activity and changes in pupil size can help to predict subjective emotional experience [54], especially positive and negative emotions [55,56,57,58]. Previous studies [57,58] found that the differences in eye movements, pupil dilation and pupil invisibility are cues for recognising positive and negative emotions.

Moreover, as an objective measure of emotions, several studies investigated skin conductivity and peripheral body temperature changes and variation for the task of detecting emotions [59,60,61]. Several signals have been investigated in [62], showing strong relation between Skin Conductivity Level (SCL) and temperature with different emotions. Even though changes from these signals could only indicate arousal and valance, the patterns from these signals together could lead to a recognition of a specific emotion. For example, a rise in heart rate accompanied by increased body temperature indicates anger, but when accompanied with a decreased body temperature, indicates fear [63].

The brain wave signal has been investigated to map brain activity with emotional responses as an objective measurement. However, brain wave analysis for emotion recognition is still a relatively young field. Most emotion recognition approaches based on brain waves were performed as positive vs. negative emotion [64,65,66], with relatively good results. On the other hand, a few studies investigated multiclass emotion classification from the brain signal [67,68].

To test this research hypothesis, the relationship between cultural acceptance and elicited emotions from video clips is evaluated and validated. We evaluate the ability to elicit target emotions from some of the English video clips validated in [10] on Saudi Arabian participants. In addition, we evaluate an initial selection of Arabic emotion elicitation video clips for comparison. For the purpose of validation, we use several sensors to measure the physiological and behavioural response for analysis and classification of the basic six emotions.

## 3. Method

**Figure 1:**
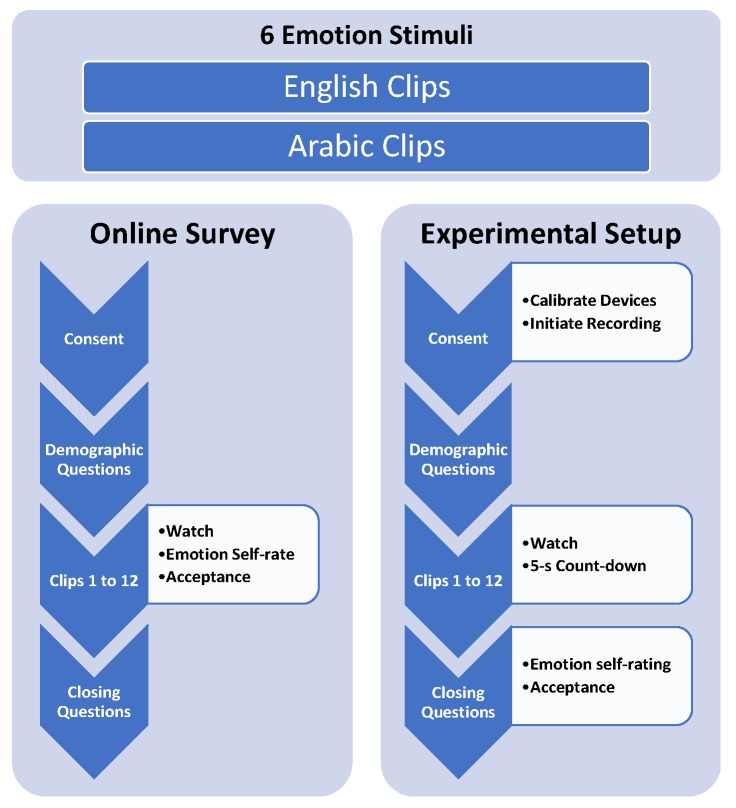
Current research methodology process.

Our work has two sub-goals: (1) measuring cultural acceptance and emotion elicitation from English clips compared to Arabic clips and (2) measuring physiological and behavioural changes during emotion elicitation from English clips compared to Arabic clips. Therefore, after selecting the stimuli, we also divided our data collection procedure into two parts (see Figure 1). For the cultural acceptance evaluation, a bigger participants sample was needed. For this purpose, we utilised an online survey. On the other hand, for measuring physiological and behavioural changes, participants have to be evaluated physically and individually in a suitable laboratory environment. For this purpose, we invited and collected data from a smaller sample of participants (see Section 3.4).

### 3.1. Stimuli

In this work, we investigate the elicitation and automated recognition of the universally-recognised six emotions (amusement, sadness, surprise, fear, anger and disgust) suggested by Ekman [18]. Therefore, we selected six video clips from the list of [10] to elicit these emotions (see Table 1). The evaluated [10] list had more than one choice for each emotion with different levels of affect. However, we selected only one clip per emotion to narrow the comparison between English and Arabic clips problem and to reduce the experiment’s total duration, which would potentially reduce subject’s boredom. Since our study focuses on cultural acceptance, we selected the most likely accepted clips that adhered to the guidelines in [35] from the clip list in [10] (as discussed in Section 2.2). Therefore, our clip selection was not always choosing the highest emotional self-rate acquired from the previous study of [10]. Even though exposing the participants to multiple video clips might discard possible bias, increasing the number of clips will increase the total time each participant spends on the questionnaire. The length of a survey has been linked to the response rate, where a shorter survey length been associated with a higher response rate [69]. Nonetheless, a data collection that contains several sessions could benefit from increasing the number of rated clips, as discussed in [15]. For example, the amusement clip, “When Harry Met Sally”, which contains sexual hints, has a stronger amusement affect than “Bill Cosby, Himself” in Western culture according to [10]. The conservative culture of Saudi Arabia does not condone showing any clip with sexual references. Therefore, the choice of “Bill Cosby, Himself” is more appropriate in this situation, even though it contains some words that might be seen in Arab culture as disrespectful to a religious figure. We acknowledge that might raise a question of objectivity in relation to the question it investigates. However, initial subjective selection of emotion stimuli is commonly performed in most emotion stimuli collection studies (e.g., [10,25,28,70]), as discussed in Section 2.1.

Dubbing the English video clips might be beneficial to reduce the speed variability of subtitle reading between subjects. However, it could lose the emotional tone from the original speech unless done professionally. Therefore, all English clips used in this study had Arabic subtitles (Subjects had different levels of English comprehension, which was not further assessed at the time, but was noted as an area of analysis in the future). Moreover, a known limitation of using subtitles is that subtitles (text [71]) could also elicit emotion. However, to reduce the effect of eliciting unexpected emotions from the subtitles, the subtitles used in this study were direct translations of what was being said with no semantic interpretation. Furthermore, subtitles were used consistently across all stimuli with English content. Nevertheless, foreign media in Saudi Arabia is popular, and the Saudi population is accustomed to watching media with different languages accompanied by subtitles [72]. Furthermore, based on our results, as can be seen in the following sections, 88% of the participants watched foreign media. This could reduce the effect of media presentation differences, since it does not conflict with the participants’ expectations. The influence of subtitles in the elicitation was not investigated and could be an area of analysis in future studies.

In addition to the English clips from [10], an initial selection of Arabic clips has been made, which is also described in Table 1. The primary aims for selecting Arabic emotion elicitation clips were to compare the cultural acceptance and to evaluate the emotion elicitation level from both English and Arabic clips. The duration of the emotion elicitation video was one of the most important factors, due to its effect on the emotion latency, rise time and offset. As discussed in Section 2.1 and based on the studies that collected and validated emotion elicitation clips, a short 1- to 2-min clip was desirable for this purpose. Therefore, in this research, a small pool of video clips (n=22) was gathered by the project team members, with an average duration of 2 min. It is worth noting that the final Arabic clips were selected based on the highest number of votes by a group of native Arabic speakers (*n* = 13). Moreover, this list was an initial step, which should be refined in future studies, since the aim of this paper is not to collect validated stimuli. An advanced method to evaluate and validate a bigger pool of Arabic clips was, for example, proposed in [14,15]. We selected one clip for each emotion (similar to English clips), where each clip had at least 80% of the votes (no inter-rater reliability was performed). This resulted in the selected Arabic clips being different in time duration from the English clip, but they were not significantly different (p=0.35).

### 3.2. Online Survey

While noting potential demographic sampling disadvantages, online surveys offer many advantages over traditional surveys, such as reduction in time and cost and reaching more subjects who otherwise would be inaccessible [73]. To get access to a larger population, we published an online survey of several pages. The survey was published using King Saud University advertisement media (e.g., mailing lists, Twitter account), student and staff boards, social media (e.g., Twitter, Facebook, etc.) and among relatives and friends of the team members. Details about the online participants can be found in Section 3.4.1. Moreover, the online survey language was in Arabic (no dual language was offered), and therefore, only Arabic-speaking participant were able to fill in the survey. The first page explained the aim of the survey as: “measuring the effect of different media on emotion elicitation and the effect of cultural background (as Saudis) for accepting such elicitation”. It also explained the method of the survey, where participants were asked to watch 12 video clips, then would answer questions after each clip. Table 2 summarises the survey pages and questions. Subjects who wished to participate checked a consent check box, which led to the remaining pages of the survey. Demographic questions were asked first before commencing to watch the clips, such as general biologic and health questions, as well as habits of watching different media. After answering the demographic questions, the next 12 pages played the selected emotion elicitation clips individually. Participants would go through the survey and watch the video clips in their own environment (home, work, etc.).

Each emotion elicitation clip was played automatically. No playback tools (e.g., pause, replay) were available in order to reduce and control variability between participants while watching the clips. Participants were not informed about the specific type of emotions under investigation, but only as a general statement that it was an emotion study (as described on the survey aim page). After watching each clip, several questions were asked on the screen about the elicited emotions felt by the participant. The term “felt;’ was described on the survey’s first page and as:“Please rate the emotions you actually experienced during watching the clips and not the emotions that you think you should feel”. Furthermore, questions about the participant’s acceptance of each clip, including listing reasons for non-acceptance of part of the clip, were also asked after watching each clip.

The presentation order effect could be controlled by providing distracting or natural exercises or by counterbalancing using block randomisation or randomising the the order [74]. The counter-balanced presentation could reduce the effect of carryover. For emotion elicitation, researchers have presented the clips in random order. However, we believe that a random order introduces variability from the affect of the previous clip, especially that these emotions have different arousal and valance. This is based on the participants’ feedback from our previous data collection [75], where participants expressed a negative experience and annoyance from the emotional fluctuation. Therefore, we chose to show each emotion for both languages consecutively to reduce the emotional fluctuation between two different emotions. We hypothesise that it would show better feeling transition, understanding and recognition of the participants’ emotions. For example, showing a sadness elicitation clip followed by an amusement elicitation clip may not give enough time to overcome the sadness emotion and might be annoying to the participant. Therefore, we showed the video clips in a particular order, while providing a 5-s countdown black screen between each clip to clear their mind, as well as providing the questionnaire questions to rate the clip.

For each emotion, first the English, then the Arabic clip were played. The emotions were in the following order: amusement, sadness, anger, fear, surprise and disgust. Even though the fixed emotion order aimed to reduce the history-affect and emotional fluctuation, it would have been preferable to randomise the English and Arabic clips’ order per emotion. However, this was not considered during the experiment execution and is acknowledged as a restraint that might introduce bias and the history-affect over the set pairs. Experiments with a larger sample and randomised stimuli to avoid amplifying congruent target emotions are planned as part of the larger scope of this project. Nonetheless, the 5-s countdown black screen and answering the questionnaire could reduce the additive effect and therefore could reduce the significance of the unavoidable order effects.

Furthermore, the time to respond to the questions after watching one clip provided some period for neutralising the elicited emotions. Moreover, we chose to show disgust elicitation clips last to reduce the disgust feeling while watching the other clips, in case the disgust feeling lasted longer than expected.

**Figure 2:**
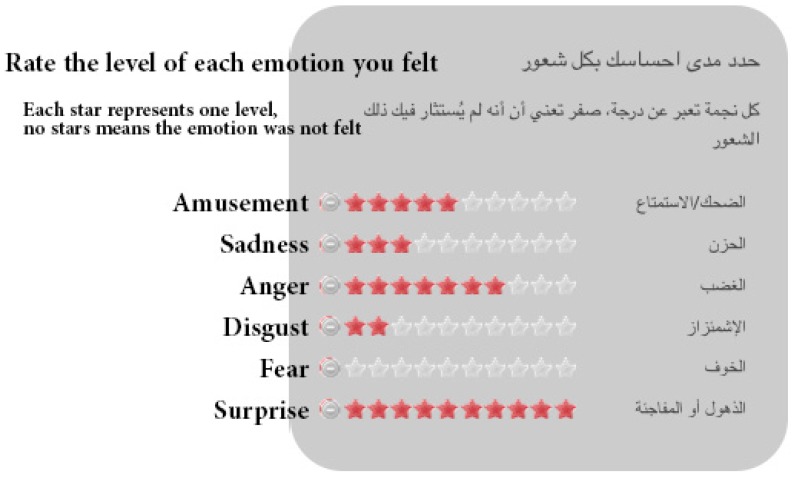
An example of rating emotions after watching a clip.

After watching the clip, participants were asked a few questions following a modified version of Gross and Levenson’s (1995) questionnaire for elicited emotion self-assessment [10] (see Table 2). Participants were asked to rate the emotions they actually felt and not the emotions that they were supposed to feel. For each of the six emotions, participants rated the level of each elicited emotion using a modified Likert scale ranging from not felt (0) to felt very strongly (10) [76]; see Figure 2. The rating scale was a modified version of the one in [10], where they used an 8-point scale (0 none; 8 strong). We chose 11 points following standard psychological rating (e.g., quality of life, pain, etc. [77,78]), where 0 is none and 10 is the highest possible). Thus, the chosen scale was: 0 not-felt, 1 weakly-felt, 4 moderately-felt, 8 strongly-felt, 10 very strongly-felt. Finally, the last page of the questionnaire asked if the participant was accompanied by another person while watching the video clips. Furthermore, we asked if the other person’s presence had any effect on the participant’s response to the questionnaire. These questions were asked to exclude participants’ ratings who were influenced by their company to avoid external distractions.

Acknowledging the importance of performing such studies in a laboratory setting, because of the important implicit effect of context on emotion induction (e.g., being accompanied by others), and to have a better control over such effects, we performed a controlled laboratory experiment. In the laboratory experiment, we used both self-report (similar to the online survey), as well as physiological measures using sensors. This laboratory experiment would potentially help in cross-validating the online survey results.

### 3.3. Emotional Response Experimental Setup

#### 3.3.1. Hardware, Software and Recording Environment

**Figure 3:**
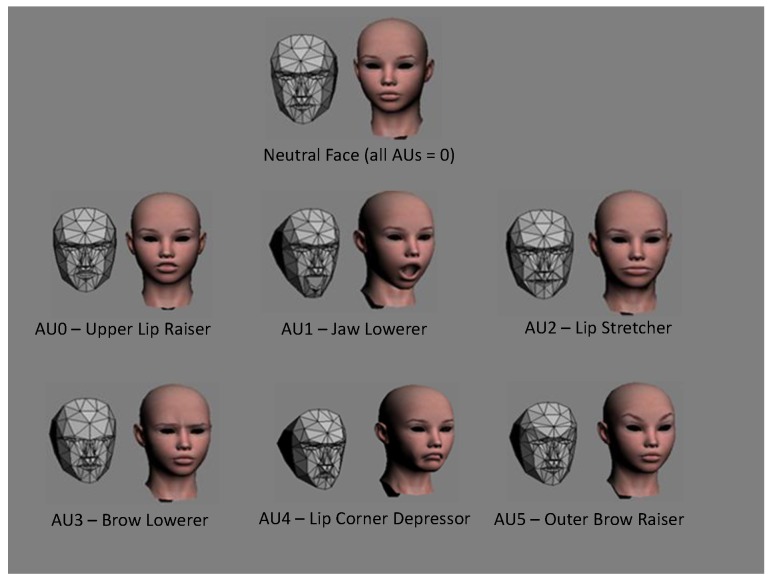
The estimated AUs from Microsoft Kinect SDK.

In this work, we used different devices to analyse and detect emotion expressions elicited by English and Arabic video clips and compared the differences. The devices used in this work are listed in Table 3, which also shows the extracted features from each device. We used the Microsoft Kinect device and its Software Development Kit (SDK) to locate and track 122 facial landmarks to estimate several AUs around the lips and eyebrows (see Figure 3), as well as the head pose. As an extension of the previous studies, we used a Tobii X120 eye tracker to evaluate eye activity, pupil dilation and gaze behaviour to recognise the basic six emotions. In addition, we used the Affectiva Q wrist-worn sensor (Q-sensor) to analyse the measured SCL and temperature as a response to emotion stimuli. Even though measures from finger or foot sensors proved to produce robust measures compared to other body locations [79], we used the wrist measures following the emotion detection literature (e.g., [59,60,61]) and also since the wrist sensor was more natural, similar to wearing a watch, compared to foot or finger sensors. In the data collection stage, we used the Emotiv Epoc headset device to record the Electroencephalogram (EEG) signal from 14 electrodes. However, in this work, the EEG signals were not analysed due to missing signal recordings and technical connectivity issues.

**Figure 4:**
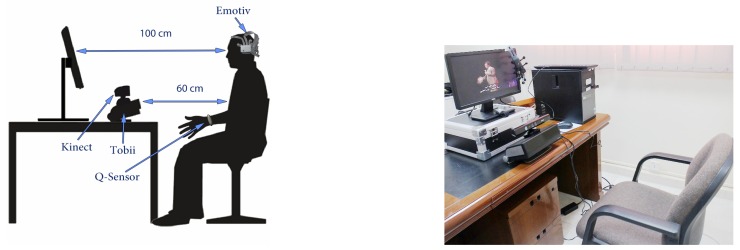
Recording setup and environment.

For the physiological and behavioural response data collection purposes, software was implemented to integrate the bio-signal devices (Emotiv, Tobii and Kinect) and record their raw data output for each subject. The software synchronises the output raw data from these devices and automatically segments the recorded data according to the clip being watched. The screen resolution, as well as the distance from the screen and the recording devices were fixed in all sessions. Although we had limited control over illumination in the recording room, we normalised the extracted features for each segment of each participant to reduce the light variability coming from the video clips themselves and the room light. The chair used in the recording environment had no wheels to prevent movement and to ensure fixed distances between the participant and the devices. Figure 4 shows the recording environment.

#### 3.3.2. Data Collection Procedure

Bio-signal and behavioural data collection followed a similar process as the online survey (see Figure 1). Consent and also a general demographic questionnaire (as in the online survey) were obtained prior to enrolling subjects in the study. Subjects were briefed about the study and were tested individually. Before the beginning of the experiment, the subjects were instructed that they were going to watch 12 clips, half of which were in English with Arabic subtitles and the other half in Arabic. They were asked to watch the video clips as they would normally do at home and were told that there would be some questions to answer afterwards about each clip and about the feelings it evoked. Each device was calibrated and checked first; upon successful calibration, the recording was started. In particular, a 5-point calibration procedure for the eye tracking device was followed prior to the experiment. To reduce large body and head movements, participants were advised to comfortably sit in the chair (see Figure 4). For the EPOC Emotiv device, all 14 channel were checked for connectivity prior to the recording.

While each participant watched the clips, the interviewer left the room to reduce distraction and to allow the participant to freely watch the clips. The software automatically showed the video clips in the same order as in the online survey and saved the raw data from the sensors while the clip was playing. A 5-s countdown black screen was shown between video clips to clear the subject’s mind. After the recording session, a post-questionnaire was asked as an interview. Moreover, to validate the induced emotion from the clips, participants were asked to rate the emotional effect of each clip on an 11-point scale as: none (score: 0) to extremely strong (score: 10). Since we were investigating several emotions (6 basic emotions), a scale of not-present/neutral to strong for each of the selected emotions is commonly used in most discrete emotion studies. Once the recording was over, subjects were thanked, and no incentives were given.

### 3.4. Participants

#### 3.4.1. Online Survey Participants

The total number of participants who gave consent for the online survey was 975, 84% of which were females, 70% from the central region of Saudi Arabia, and their age ranged from 18–66 years (μ=26.4y,σ=7y). To address the gender imbalance and to analyse the gender differences, gender groups’ results were analysed separately, as discussed in the following sections. However, the age groups were not analysed separately due to the small sample of participants in the older age groups (around 7% older than 35 y with only 1% older than 50 y), where the influence of age groups was minimal. Due to physical and/or mental health issues, some participants (7% and 3.4%, respectively) were not allowed to complete the survey. This was done to not effect their condition in case of a very sensitive or intensive emotion stimuli. Moreover, due to technical issues, such as mobile phone incompatibility and Internet connection, only 345 participants were able to play the videos and complete the survey. Having 12 video clips with an average duration of 2 min, the entire survey took about 40 min on average to complete. Only 166 participants finished the entire survey. However, for the analysis, we used the available ratings for each clip regardless of whether the participant finished the full survey. To address the response imbalance in each clip, we used the weighted average of ratings for each clip in the evaluation. The average number of respondents for each clip was 220. For more details, Table 4 shows the number of responses for each video clip.

Most participants (91%) preferred subtitled over dubbed video clips when watching foreign media, which supports our choice of subtitled video clips rather than dubbed ones. Surprisingly, while 20% of participants never watched Arabic media, only 12% of them never watched foreign media, which indicates the popularity of foreign media in Saudi Arabia. Investigating the effect of another person’s presence while watching the clips, 93.5% of the respondents were alone, and only 4.5% were influenced by company while answering the questions.

#### 3.4.2. Physiological and Behavioural Measurement Experiment Participants

We collected bio-signals from 29 subjects while watching the selected emotion elicitation clips. None of the participants had a physical or mental disorder. The participants’ age ranged from 18–45 years. Out of the 29 subjects, 59% of the participants had normal vision, and the rest were using either glasses or corrective lenses. While a larger cohort would be preferable, we believe that the current sample size was sufficient for the purpose of validating the results from the online survey, where the two cohort were completely separate.

### 3.5. Data Preparation

The recorded sessions containing signals from all devices needed to be prepared for the classification phase. This preparation included segmenting the sessions for each device and each clip, as well as giving a label (ground truth) to each segment for the classification problem. For each segment, the ground truth was the participants’ self-rating of the elicited emotion of each clip. However, each clip could elicit different emotions than the target emotion or a combination of emotions, which introduces the high dimensionality classification problem. This current study investigated the cultural aspect in relation to eliciting the target emotion from each clip. Therefore, segments from each device and for each clip were selected based on the participant self-rating for the target elicited emotions during watching each clip. That is, if the participant felt the target emotion, the segment would be used because the stimulus elicited the emotion, otherwise it would be discarded. In other words, to reduce the complexity of the classification problem, the self-rating ground truth of the segment was cross-referenced with the target emotion. Nevertheless, it would be an interesting future research direction to study participants’ physiological and behavioural responses in comparison with their self-rating for each stimulus.

Moreover, for technical reasons, such as an occluded face and/or eyes, failing to record timestamps or device disconnection, some segments were not recorded, therefore not selected for this analysis. Table 5 shows the number of segments used in this study for each device and for each emotional clip.

The extracted raw data from each device (Emotiv, Tobii and Kinect) were automatically synchronised and segmented for each subject and each clip using the software implemented for this purpose. The synchronisation was handled by starting the signal recordings at the same time with a click of one button and also attaching a common timestamp to the signals from each device (Emotiv, Tobii and Kinect). However, the Q-sensor was not attached to the computer nor to the software. Therefore, to segment its raw data for each subject and each clip, we used the timestamp in the Q-sensor raw data and then matched it with the recording timestamp from our software, which was performed offline, using a MATLAB program.

### 3.6. Feature Extraction

**Figure 5:**
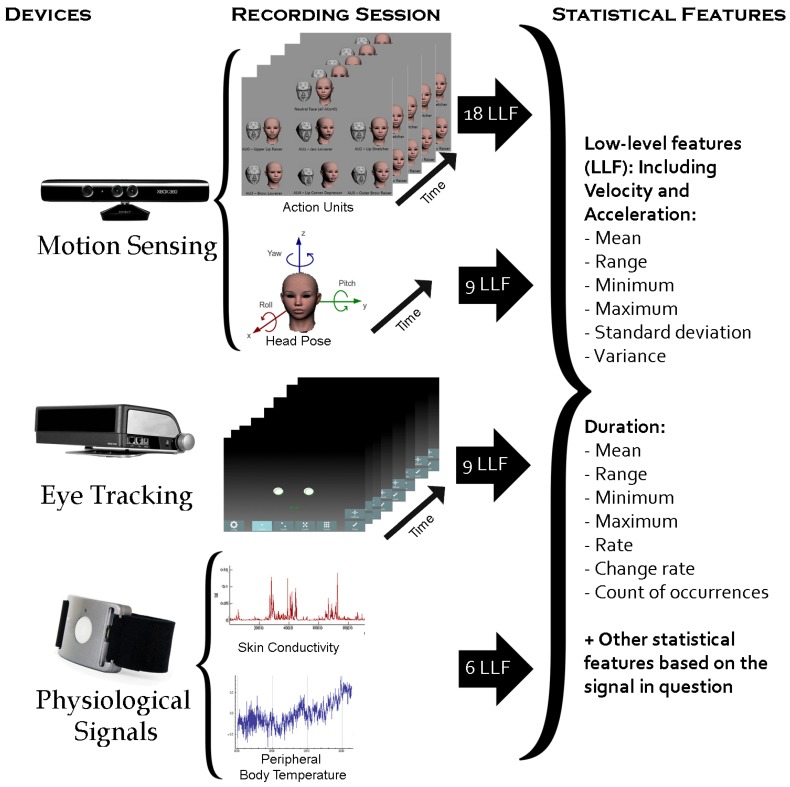
Summary of feature extraction procedure.

To recognise patterns of subjects’ emotional responses, we extracted statistical features from facial expression, head movement, eye activity and skin conductivity. The process of feature extraction is summarised in Figure 5 and described in detail in the following subsections.

#### 3.6.1. Facial Expression Features

The Microsoft Kinect SDK locates and tracks 122 landmarks in the face, which can be used to extract specific AUs or measure subtle changes in facial muscles. This SDK also extracts 6 AUs that represent some facial muscle activity, mainly on the lips and eyebrows (see Figure 3). For simplicity, we used the intensity of the extracted 6 AUs in each frame (30 frames per second), which are already normalised from −1–1. For each frame, we also extracted the velocity and acceleration of changes for each of these AUs, which gave a total of 18 low-level features for each frame. Over each segment (each clip being watched by each participant), we calculated 18 statistical features for each AU and their velocity and acceleration, which were:

- Mean, range, minimum, maximum, standard deviation and variance for all 18 low-level features (18×6).
- We also calculated several statistical duration features such as maximum, minimum, range and average of the duration, as well as its rate to total duration and count of the occurrence of:
  - each AU (6 features × 6 AUs),
  - fast changes of each AU (6 features × 6 AUs),
  - slow changes of each AU (6 features × 6 AUs),
  - continuous changes of each AU (6 features × 6 AUs),
  - steady state of each AU (6 features × 6 AUs).

#### 3.6.2. Head Pose Features

Using the Microsoft Kinect SDK, the yaw, pitch and roll angles of the head pose were recorded in each frame (30 frames per second). Once these three angles were extracted for each frame, their velocity and acceleration were calculated to give in total 9 features per frame. Using these 9 low-level features, a total of 184 statistical features (“functionals”) were calculated over each segment, which were:

- Maximum, minimum, range, mean, variance and standard deviation for all 9 low-level features (6 × 9 features)
- Maximum, minimum, range and average duration of head direction: left, right, up and down, tilting clockwise and anticlockwise (4 × 6 features)
- Head direction duration rate and rate of different head directions for the non-frontal head direction for all directions mentioned above (2 × 6 features)
- Change in the head direction rate for all directions mentioned above (1 × 6 features)
- Total number of changes of head direction for yaw, roll, pitch, as well as the total number of these changes (4 features)
- Maximum, minimum, range, mean, variance, duration and rate for slow, fast, steady and continuous movement of yaw, roll and pitch (7 × 3 DOF × 4 features)

#### 3.6.3. Eye Activity and Gaze Features

Excluding frames where the eyes were not detected by the eye tracker, we calculated 9 features per frame (30 frames per second) of raw data extracted from the Tobii eye tracker, as follows:

- Distance between eye gaze point positions from one frame to the next for each eye and its speed (Δ) and acceleration (ΔΔ) were calculated to measure the changes in eye gaze points. The longer the distance, the faster the eye gaze change (2 × 3 features).
- The difference between the distance from the left eye to the eye tracker and the distance from the right eye to the eye tracker were calculated, to measure head rotation approximately (1 feature).
- Normalised pupil size for each eye, to measure emotional arousal (2 × 1 features).

Over the low-level features mentioned above, we calculated 147 statistical functional features to measure the pattern of eye activity. Those features were:

- The mean, standard deviation (std), variance (var), maximum, minimum and range for all low-level features mentioned above (6×9).
- Even though blink features were not available from the Tobii X120, we measured the absence of the pupil in the video frames. Absence of the left pupil only indicates left head rotation, and vice versa. Absence of both pupils could represent blinks, occluded eyes or head rotation being out of the eye tracker’s range, such as extreme looking up/down or left/right. We measured the mean, standard deviation (std) and variance (var) of the absence of left, right or both pupils (3×3)
- We also calculated several statistical features such as maximum, minimum, range and average of the duration, as well as its rate to total duration and count of the occurrence of:
  - fast and slow changes of eye gaze for each eye (6×2×2 eyes);
  - left and right head rotation (6×3);
  - large and small pupil size for each eye (6×2×2 eyes);
  - the absence of left, right or both eyes (6×3).

#### 3.6.4. Skin Conductance and Temperature Features

The Q-sensor wrist band measured the 3D location of the hand, as well as peripheral body temperature and skin conductance with a sampling rate of 32 Hz. In this work, we extracted statistical features from the peripheral body temperature and skin conductance over each segment, given that each clip being watched by each subject counted as a segment. For each sample (frame), we also extracted the velocity and acceleration of changes of temperature and skin conductance, which gave a total of 6 low-level features for each frame. Over each segment, we calculated 108 statistical features for temperature and skin conductance and their velocity and acceleration, which were:

- Mean, range, minimum, maximum, standard deviation and variance for each of the 6 low-level features (6×6 features).
- We also calculated six statistical duration features such as maximum, minimum, range and average of the duration, as well as its rate to total duration and count of the occurrence of:
  - high temperature and skin conductance (6×2).
  - low temperature and skin conductance (6×2).
  - fast changes in temperature and skin conductance (6×2).
  - slow changes in temperature and skin conductance (6×2).
  - continuous changes in temperature and skin conductance (6×2).
  - steady state in temperature and skin conductance (6×2).

The above duration features were detected when the feature in question was higher or lower than a threshold. The threshold was determined as the average of the feature in question plus/minus the standard deviation of that feature for each segment. Moreover, the fast and slow changes were calculated from the velocity low-level features based on the threshold. That is, when the difference between two frames was higher than the threshold, a fast change was detected, and vice versa for the slow changes. The same applied for the continuous changes and the steady state, which were calculated from the acceleration low-level features. In this work, continuous change was the continuously high difference in velocity. For example, a continuous head movement was when the head was continuously moving (changing direction or speed). On the other hand, steady state was when there was no or low velocity (e.g., no change in direction or speed of head movement).

### 3.7. Analysis

#### 3.7.1. Statistical Analysis

For the online survey, rated target and non-target emotions were compared statistically for significance. Moreover, we compared the significance between target emotion elicitation between English and Arabic clips. For each clip, we conducted a two-sample two-tailed *t*-test for the target vs. each of the non-target emotions with Bonferroni correction for multiple comparisons. In addition, we conducted a two-sample two-tailed *t*-test for the English target vs. Arabic target emotion for each clip. The two-tailed *t*-tests were obtained assuming unequal variances with significance p=0.05.

In order to characterise the basic six emotions from the physiological and behavioural measures, the extracted statistical functionals from each device for each emotion were compared. A one-way between groups analysis of variance (ANOVA) test was used for this purpose. In statistics, the one-way ANOVA is used to analyse the statistically-significant differences between the means of a factor in three or more independent (unrelated) groups. In our case, one-way ANOVA was selected to analyse the statistical significance differences of each extracted feature (factor) for the six emotions (groups), with significance *p* = 0.05. This analysis will help determine which features had an influence on differentiating between emotions. The sample size for each emotion and device is shown in Table 5.

#### 3.7.2. Classification Methods

For the classification experiments reported in this work, Support Vector Machine (SVM) classifiers were used. SVMs are discriminative models that learn boundaries between classes. To increase the accuracy of SVMs, the cost and gamma parameters were optimised. LibSVM [80] was used to this end with a wide range of grid search for the best parameters with a Radial Basis Function (RBF) kernel. The classification was performed as multiclass (6 emotion classes) in a subject-independent scenario. As SVM mainly discriminates binary classes, the multiclass classification could be performed as one-verses-all, where each SVM separates a single class from all remaining classes, or as one-versus-one, where each SVM separates a pair of classes. In this work, we used the one-versus-one approach, where the final class is the one selected by the majority of the classifiers. Moreover, to mitigate the effect of the limited amount of data, a leave-one-out cross-validation was used without any overlap between training and testing data.

Normalising the data to the same range ensured that each individual feature had an equal chance of having an effect on the classification problem. When modelling inputs with different scales, normalisation is recommended [81], which was the case in our study. In this work, Min-Max normalisation was performed, which scales the values between 0 and 1. Min-Max normalisation has the advantage of preserving exactly all relationships in the data, even though it does not reduce the effects of outliers. In addition, the ANOVA test (see Section 4.2.1) was used as a feature selection. That is, only features that passed the significance group ANOVA test with p≤0.05 in the training set were selected, and then, these features were selected in the testing set. Acknowledging the variety and advancement in the feature selection method, ANOVA was chosen for its simplicity in feature dimension reduction. Moreover, the aim of this paper was not to investigate classification results’ enhancement, rather to compare stimuli responses. The final selected feature vectors were labelled with the target emotions, using only subjects that actually felt the target emotion based on their self-rating questions (see Section 3.5 and Table 5 for more details).

## 4. Results

### 4.1. Survey Results

#### 4.1.1. Cultural Acceptance of the Clips

**Figure 6:**
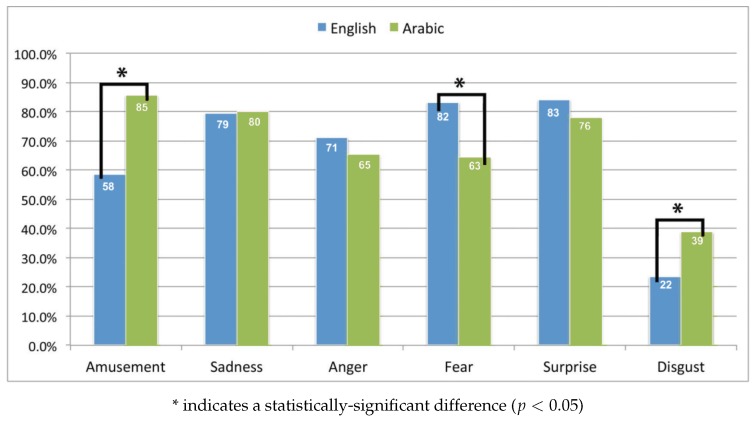
Cultural acceptance percentage for each of the emotion elicitation clips.

The goal of this study was to see the effect of cultural acceptance on induced emotion from video clips. A “yes/no” and an open answer question in the survey investigated the cultural acceptance for each clip. Respondents were asked of their general acceptance for each clip, as well as indicating if anything in the clip had annoyed them. Figure 6 shows the acceptance as a percentage for each clip selected in this study (note that the number of respondents for each clip is shown in Table 4). Table 6 shows the main reasons that annoyed respondents, which led to not accepting a clip.

As can be seen, regardless of the clips’ language and origin, a majority of the video clips (seven out of 12) had more than 70% cultural acceptance. Comparing clips’ language and origin, half of the emotions in question, in particular sadness, anger and surprise had similar cultural acceptance for both English and Arabic clips. The other half of elicited emotion clips, namely amusement, fear and disgust, had significantly different acceptance between English and Arabic clips. Interestingly, the English clip that elicited fear had a significantly higher acceptance rate than the Arabic one, but it was the other way around for amusement and disgust emotion elicitation clips. This finding suggests that the origin of the clips was not a factor for the cultural acceptance, as the respondents objectively accepted or rejected clips from both English and Arabic lists based on the content. This finding contradicts the Chinese prescription of Western culture, where it was found that participants’ prescription was affected by their own background [82]. The reasons for rejecting the video clips were influenced by the cultural and religious background of the respondents, as listed in Table 6.

The English clip that induced amusement was not accepted by over 40% of the respondents, mainly due to curse words, especially using Jesus’ name as a curse word, which is considered as disrespecting a religious figure in the Islamic religion [83]. As the amusement elicitation clip showed Bill Cosby criticising his father and childhood, 42% of respondents explained that this act was disrespectful to the parents, which is considered a sin in Islamic principles [84]. The same rejection reason was stated by 56% of participants about the Arabic amusement elicitation clip, where the female actor was talking loudly to her mother.

The Arabic clip inducing anger was rejected by over 45% of the respondents, mainly for depicting companions of the Prophet Muhammad, which is prohibited by most Islamic scholars [85]. In their view, converting a book to a movie will require editing and adding to the main script, which will compromise authenticity [85]. Besides, the appearance, personality and behaviour of the actors are not comparable with the highly respected lives of the religious figures [85].

The Arabic clip inducing fear was rejected by over 46% of total respondents, mainly for the action of attacking a woman by two men, which is not accepted in most cultures. Both Arabic and English video clips that induced disgust were highly rejected mainly for the intensity of the disgust felt.

Comparing the gender differences in the clips’ acceptance, the results showed no significant differences in the acceptance between the two groups of participants in most clips from both languages. One exception was the disgust clip in both languages, where the results showed significantly higher acceptance in male participants compared to female ones. The difference between the two genders in accepting the clips might be explained by the fact that women have higher disgust sensitivity than men [86]. This also supports that the low acceptance of these clips was based on the intense feeling of disgust, and not for cultural reasons. Moreover, the reason for high acceptance from men might be based on the emotion self-rating of these clips, where male participants self-rated the disgust clips as amusing, as will be discussed later.

As can be seen, certain values of the Islamic religion and culture have a high effect on accepting or rejecting a clip, regardless of the clip’s origin or language.

#### 4.1.2. Emotion Elicitation Rates of the Clips

**Figure 7:**
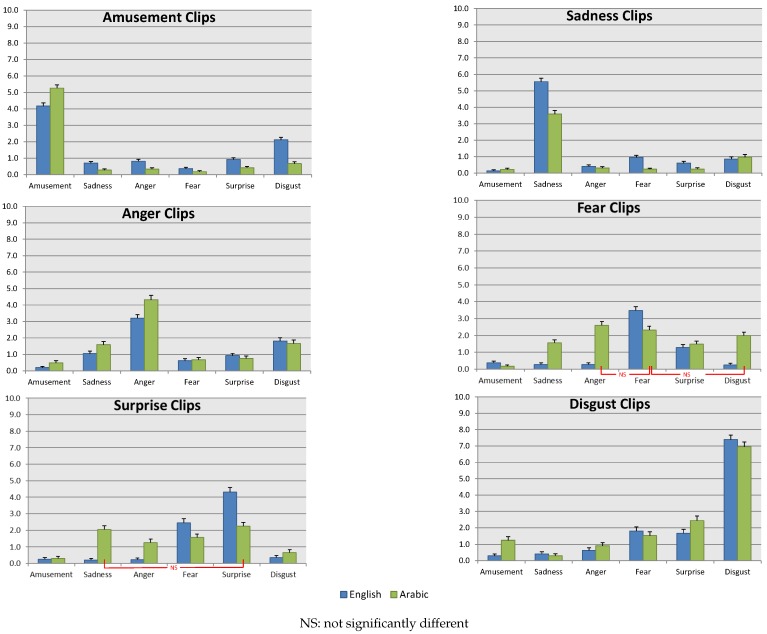
Emotion elicitation average self-rate and its standard error for both English and Arabic clips.

As mentioned in Section 3.2, after watching each clip, participants were asked to rate all emotions they actually felt (from a list of six prototypical emotions) and not the emotions that they were supposed to feel. Moreover, participants could choose more than one emotion class (see Figure 2). For each clip, participants rated the intensity of each elicited emotion (zero not felt, 10 felt strongly). Since the participants rated the intensity of emotions they felt from 0–10, the average rating of each emotion in each clip from all participants was calculated. Due to the differences in the level of intensity per emotion per clip for each participant, the proportion of the targeted emotion compared with non-target emotions expressed by the participants was not calculated. Figure 7 shows the average rate of elicited emotions from each clip.

Regardless of the cultural acceptance and the average rate of each emotion, all English clips were significantly able to induce the target emotion compared to each of the non-target emotions. However, only sadness and disgust elicitation clips had an above average (more than five points out of 10) self-rate of eliciting the target emotion. The low self-rate of the elicited target emotion for the remaining video clips was not correlated with the cultural acceptance. For example, the fear and surprise elicitation clips had a high acceptance rate (82%), yet the average self-rate of eliciting the target emotion was only around four points. On the other hand, the amusement elicitation clips had a low acceptance rate (58%), yet the average self-rate of eliciting the target emotion was also around four points.

Most of the Arabic video clips were significantly (statistical significance) able to induce the target emotion compared to each of the non-target emotions, with the exception of fear- and surprise-inducing clips (as indicated by “NS” in Figure 7). For instance, the fear inducing Arabic clip elicited both anger and disgust. The reason behind the mixed emotion elicitation, we believe, is the low acceptance from the respondents. Respondents reported anger more than fear, as well as disgust, based on rejecting the content of the clip where two men attacked a woman. The surprise elicitation Arabic clip induced a high self-rate of sadness as well. The surprise clip shows a man being shot (see Table 1), which might be the reason for inducing sadness for the victim’s sudden death. As with the English clips, only two Arabic clips (amusement and disgust) had an above average (more than five points) self-rate for eliciting the target emotion.

Comparing Arabic and English emotion elicitation clips statistically showed that each pair of video clips was significantly different from each other in eliciting the target emotion. In most cases, with the exception of amusement and anger, the English video clips had a statistically higher rate of emotional elicitation than the Arabic ones. For the amusement elicitation clips, the English clip had a lower cultural acceptance compared to the Arabic one, which could explain the lower rate of amusement in the English clip. When it comes to humour, jokes are more effective with shared social values, norms, etc. [87], which make inducing amusement more effective when using video clips from the same cultural background. Even though the Arabic clip that induced anger was not culturally accepted, it evoked a higher rate of anger compared with the English clip. The reason might be that the anger was induced by rejecting the content of depicting religious figures rather than the scene itself, which should be considered in future clip selection.

**Figure 8:**
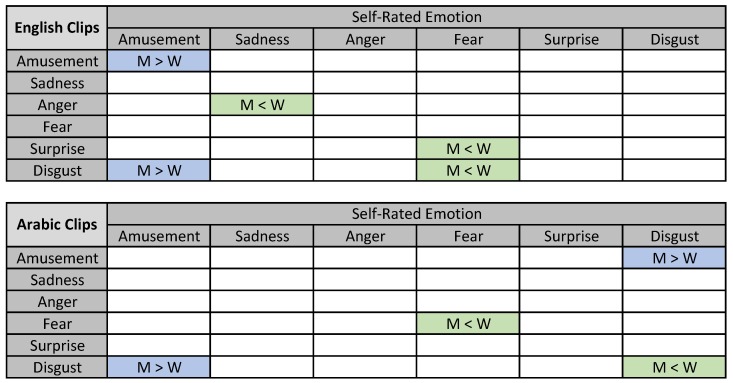
Significant gender differences in self-rated induced emotions from each clip. Using corrected *t*-test, empty cells indicate no significant differences between the reported emotions.

Studies showed strong gender differences in emotional evaluation and expression, which proved to be culturally independent [88]. Therefore, we explored gender differences in emotion self-rating while watching the clips. The results are presented in Figure 8, where only statistically-significant differences are shown (using the corrected *t*-test for multiple tests). Based on the results, women showed higher self-rated sadness and fear compared to men, which was expected based on gender-differences in emotion studies [88]. The studies showed that women were more expressive for weak emotions (e.g., fear, sadness), while men showed strong emotion (e.g., anger). Moreover, women self-rating for the Arabic disgust clip differed between the genders, where women rated higher disgust, while men rated higher amusement. Studies have shown that women are more sensitive to disgust than men [86]. Surprisingly, men reported high amusement from disgust clips from both languages, yet reported disgust from the Arabic amusement clip. This finding might be culturally dependent, where more studies should focus on amusement and disgust triggers in men from Arab culture.

As described in Section 3, both online survey and emotional response experimental setups requested the participant to self-rate the emotion they experienced from each clip. It is worth noting that the self-rated emotions of each clip in the emotional response experimental setup were similar (no significant differences) to the self-rated emotion from the online survey. This could suggest that the perception of the sample in the experimental setup was aligned with the perception sample in the online survey.

### 4.2. Analysis and Classification of Physiological and Behavioural Responses

#### 4.2.1. Statistical Analysis over Extracted Physiological and Behavioural Features

To gain a better understanding of physiological and behavioural changes during the expression of emotions, the extracted features from each emotion elicitation segment were analysed statistically for differences. In particular, one-way group ANOVA tests were calculated over each individual feature, where the six emotions formed the groups. The analysis was performed over English and Arabic video clips individually to compare the two sets with each other, as well as to select the common features that were significantly different in each emotion group in both languages, which would ensure a fair comparison. Even though different combinations of features might be more representative for one clip or group of clips, using the same selected features for both English and Arabic emotional responses would reduce variability for the comparison. Future studies could investigate advanced and dynamic feature selection techniques to investigate this representation to improve the analysis and classification.

Table 7 shows the common features that were statistically significant , based on the results of ANOVA group test with significance *p* = 0.05, to differentiate emotions in both language sets. For example, all six AUs around the lips and eyebrows were significantly different not only in their values, but also their duration and movement changes, which is in line with previous action units’ investigations [89,90,91]. The speed and direction of head movement were also significant, which is expected to distinguish emotions, in line with [92]. For example, backward head movement when laughing, looking away when disgusted or a sudden head movement when surprised have different head movement patterns [92]. Moreover, the eye openness and pupil size showed significance for different emotions, which could indicate that the subjects were focused and responding to the stimuli, in line with [57,93]. In addition, as expected, based on the literature [94], the level of skin conductance (SCL) showed significant measures of changes with different emotions. Finally, as with SCL, analysing the peripheral body temperature showed significant temperature value differences and their change levels. These findings and significant features indicate the variation of subjects’ feelings induced by the video clips [94,95,96].

Since irrelevant features cause high data dimensionality and may affect the performance of the system, feature selection techniques are important to overcome this by selecting related features. There is a variety of feature selection techniques, including statistical methods, filter methods, search strategies, learning techniques, etc. In this work, we used a statistical feature selection based on ANOVA tests, where the evaluation determined the existence of differences between emotion groups for each feature. That is, only features that pass an ANOVA test with p≤0.05 in emotion groups were selected as listed in Table 7 and were used for the classifier, as explained in the next section. Moreover, having the same list of features for both English and Arabic segments is important to assure fair comparison for the classification results. It is worth noting that the number of features that were selected based on the ANOVA test were 98 features out of 727 extracted features.

#### 4.2.2. Automatic Classification of Emotions

Using an ANOVA test for feature selection and a multiclass SVM classifier, we attempted to classify six elicited emotions automatically from the English and Arabic clips. These were classified individually in order to compare the classification accuracy between the two clip sets, which was expected to give an insight to correlate cultural background with the selection of emotion elicitation clips.

**Figure 9:**
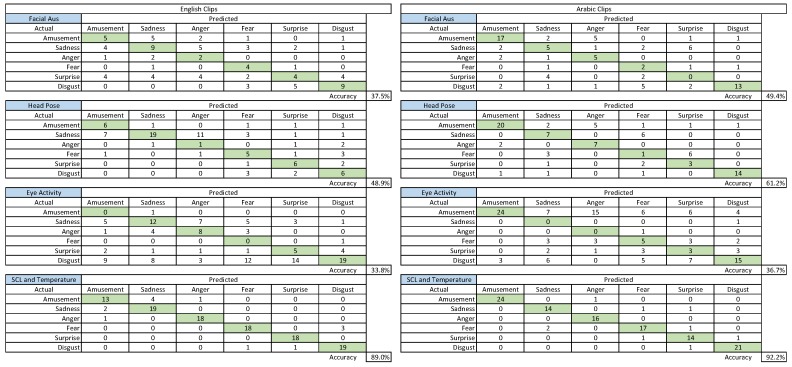
Confusion matrices for emotion classification from different sensors.

Table 8 shows the classification accuracy using a multiclass (six emotions) SVM classifier from the statistically-selected features from different physiological and behavioural signals. Detailed confusion matrices for the emotion classification are illustrated in Figure 9, which also shows the influences of each sensor on the individual emotions’ classification. Although the overall recognition rate was low, having the same method for the analysis in both English and Arabic sets ensured a fair comparison, which is the goal of this study. Moreover, the recognition rate could be improved using other advanced methods of feature selection and advanced parameter optimisation for the classification, which is not the scope of this current study, but will be evaluated in future work. Emotion recognition from Arabic video clips was higher than emotion recognition from English clips, as can be seen in Table 8 and described bellow.

Looking at the classification accuracy of physiological and behavioural signals individually shows varying results. Interestingly, SCL- and temperature-selected features gave the best automatic classification for this problem for both English and Arabic video clips compared to the other signals. Even with this primary investigation, this finding shows a high potential for accurate emotion recognition from the SCL and temperature signals, which is in line with previous studies (e.g., [97,98]). Furthermore, this finding might indicate that the SCL and temperature signals and their extracted features have distinguishing characteristics for the six emotions compared to the other physiological and behavioural signals. That is, other sensor signals could be sensitive to both physiological, behavioural, as well as technical noise. Moreover, unlike the other sensors where their features depend on estimations (e.g., estimate face AUs based on face landmarks), SCL and temperature features were in direct connect with the body, where it needs less estimation effort.

Several action units have been used to identify certain emotions [43,89,90,91]. Even though our analysis showed significant differences between emotions in a large number of extracted action unit features, this was not reflected on the automatic classification results. The low classification results from the action unit features were unanticipated compared to the literature [99,100,101,102]. This might be due to our use of statistical features of action units for emotion classification in comparison with the previous studies, where they used temporal features for the classification of emotions. Moreover, the AUs used in this work might not cover the spectrum of emotion expressions, where more and different AUs might have better recognition accuracy of the six emotions. Furthermore, using other AUs extracted from the facial points using advanced techniques could help to improve the results.

Similarly to facial action units, the eye activity features had low performance results in the classification of emotions. In previous works, eye activities showed potential in multi-emotion classification [103] and in positive-negative emotion classification [55,56,57,58,104]. However, the classification results from the extracted eye activity features were not as expected. This might be due to the eye activities being influenced by other physiological factors such as light, cognitive and attentional [50,52], which cannot be controlled when using emotion elicitation clips. The eye activity features might also be effected by technical factors such as a noisy signal, undetected eye position, out-of-range head orientation, etc.

Head pose features have been used to identify emotions [45,92,105,106], where they could be supporting cues along with other cues (e.g., facial expression) in classifying emotions. In this work, Head pose features had a moderate result, which could be useful once fused with other features.

Moreover, in the physiological and behavioural data collection, self-rating of elicited emotions was also collected in the post-questionnaire, where they were similar to the self-rating of emotions from the online survey (illustrated in Figure 7). It is worth noting is that the English and Arabic self-rating of elicited emotions was not significantly different, yet the classification accuracy was higher using Arabic elicitation clips. This finding hints that using video clips selected from the participants’ culture is more likely to induce a higher rate of the target emotions.

## 5. Discussion

In order to investigate the relationship between cultural acceptance of emotional stimuli with their emotional response in Arab culture, several objectives were derived. First, we evaluated the suitability of a standardized English set of emotion stimuli from [10] in eliciting the target emotions in the Arab sample (Saudis in particular). Then, we compared the results with an initial selection of the Arabic set of clips that was designed to elicit the same set of emotions. The finding suggested that the origin and language of the clip (i.e., English vs. Arabic) was not a direct factor of eliciting the target emotion. That is, some of the English clips induced the target emotion similarly (sometimes higher) to their counterpart in the Arabic clips. This is in line with the finding from [22], where some of the emotion stimuli from [10] elicited the target emotion on Japanese participants.

Analysing the cultural acceptance of the English and Arabic clips, some of the clips from both language groups had low cultural acceptance. In line with the finding of [34,35], the reasons for clip content rejections were highly correlated with culture and religion aspects.

The main goal of this work is to examine cultural acceptance influence on eliciting target emotion form emotional stimuli. To the best of our knowledge, only a few studies exist that explored the dimension of culture in influencing induced emotions. A recent review paper investigated clips as an emotion elicitation media for the last decade, where they emphasised the lack and the importance of including culture as a factor for such clips’ validation [107]. A recent study investigated the race-ethnicity factor for inducing emotions from video clips [108], where differences in expressing emotion were found between participants. Even though their analysis of the race-ethnicity factor was limited because of the mixed race-ethnicity of the participants, it showed some differences in reported emotion that were correlated with race. The study of [22] showed that non-target emotions were highly elicited when the content of the clip contradicted with Japanese culture. The same result was also present in our study, where some of the clips from both English and Arabic clips that contained contentious content highly elicited non-target emotions.

Moreover, the results showed that some of the English clips elicited the target emotions more than the Arabic clips. The reason behind this might be that American movies dominate both in terms of commercial success and in terms of quality [109], provided that their content is culturally accepted. Having the video clips play in the order of first English, then Arabic for the same target emotion might run the risk of comparing the elicited emotion level based on the previous clip (history effect). This risk might be the reason behind the lower self-rate for Arabic video clips compared to the English ones. As mentioned earlier, randomizing the English and Arabic clips’ order could have reduced this effect not only on the self-rating of elicited emotion, but also to avoid amplifying congruent target emotions. Randomization of English and Arabic clips will be accounted for as part of the larger scope of this project.

The last objective of this work is to analyse and classify the elicited target emotions using several physiological and behavioural responses collected in the laboratory setting. The classification results varied between the sensors. Yet, the emotion classification results for the induced emotion from Arabic clips were consistently higher compared to English clips. This could suggest that clips from the participants’ culture are more likely to induce the target emotion. However, the clips should be selected with respect to the cultural guideline to avoid inducing non-target emotions and to ensure that the clips are accepted by a wide rage of participants.

The classification investigation presented in this study is primary, where advanced methods could increase the classification accuracy. For example, temporal feature extraction [110], advanced feature selection [111], and advance classification [111,112] methods could have high performing results in emotion classification. Moreover, the data size in this work was fairly small (29 subjects), where more data points could enhance the generalizability of the emotion recognition model [113]. Fusion techniques of the most effective physiological and behavioural responses could not only enhance the accuracy, but also increase the confidence in the classification results [114]. Typically, a larger number of features compared to the data points could decrease the classification accuracy [115], where choosing the optimal number of features relative to the sample size could reduce this effect [115,116]. This paper did not investigate the potential effect of the selected number of features on the classification accuracy. However, this aspect along with investigating advanced pattern recognition techniques in emotion recognition is planned for the larger scope of this project.

Analysing the validity threats of the experiment design, several external, internal and conclusion threats were identified [117]. Most of the internal validity threats for one-group experiments were accounted for in the experiment design, that is: (1) maturation: by excluding segments where participants have seen a clip before; (2) statistical regression: all participants who have felt the target emotions were included regardless of the intensity of the felt emotion; (3) instrumentation: most environmental settings (e.g., distance between devices, experiment administered by a system) were constant, except for the lights, which was later normalized before feature extraction. Nonetheless, as mentioned before, the history aspect of internal validity threats needs to be accounted for in the order of viewing the English and Arabic clips to reduce potential history effects in emotion elicitation. Regrading external validity threats, population validity was tested by comparing the self-rating from the experiment sample with the self-rating from the online sample, where the results showed that the experiment sample was representative of the population. Nevertheless, a larger sample would be preferable to study the generalisability of the emotion classification results. For the conclusion validity threats, a thorough comparison of the literature was performed, which included the differences and limitations of this study, as described above and in the Results Section.

## 6. Conclusions and Future Work

Recognition and understanding of emotions could be beneficial for different fields and applications such as HCI, education, physiological, neurobiological, advertisement and sociological studies. Several standardised approaches to elicit emotions in a replicable method have been proposed. Using movie clips to elicit discrete emotions is the most popular method, where a validated set of video clips has been made available for the purpose of eliciting the six universal emotions suggested by Ekman [10]. Given the cultural differences, the validated clips, even when dubbed, might not elicit the target emotions in different cultures. Therefore, in this paper, we evaluated and validated the English set of emotion elicitation clips in [10] for their cultural acceptance and suitability to elicit six universal discrete emotions suggested by Ekman in an Arabic sample. For comparison reasons, an initial Arabic set of video clips was also selected for the same purpose of cultural acceptance and emotion elicitation effectiveness.

In order to get access to a larger sample, an online survey was published to evaluate the cultural acceptance and emotion elicitation for the selected video clips from both English and Arabic sets. The online survey had on average 220 respondents for each clip. The results showed that origin of the clip was not a factor for accepting or rejecting the clip, as there were culturally unaccepted video clips from both languages. The main factors for culturally accepting or rejecting a clip were religious reasons and cultural values. Moreover, in most cases, cultural acceptance of clips did not show a direct correlation with the self-rate of elicited emotions, as some of the highly culturally-accepted video clips had a low self-rate of target emotions and vice versa. Nevertheless, a careful selection of the video clips is needed based on the cultural values, to eliminate variability and assure replicable emotion elicitation. An interesting example was the Arabic anger elicitation clip, where the anger emotion was elicited unintentionally by culturally rejecting the clip content rather than the scene itself.

For validation purposes, several devices were used to measure physiological and behavioural responses associated with emotional expression while watching the clips. Physiological and behavioural responses were measured from 29 subjects while watching the selected clips. A multiclass SVM classifier using the psychological measures resulted in a higher recognition rate for the elicited target emotion from Arabic video clips (avg. 60%) compared to the English clips (avg. 52%). This result might reflect that using video clips from the subjects’ culture is more likely to elicit the target emotions.

Future work will progress to carefully select video clips for eliciting emotions in affect studies on an Arab population. The selected video clips must first be validated for cultural acceptance and effectiveness of eliciting target emotions in a large Arabic sample ([10] used 494 participants). The validated video clips can be then used to design an emotion elicitation framework for Arabic speakers [14]. Moreover, future work could investigate recent deep learning methods (e.g., convolutional Multiple kernel learning (MKL) [112]) for multimodal emotion recognition, when a bigger sample of data is collected.

## Figures and Tables

**Table 1:** Description of English and Arabic video clips used as stimuli.

Emotion	Duration (s)	Description
**English Clips**		
Amusement	120	Stand-up comedy showing Bill Cosby talking about his father and his childhood. (“Bill Cosby, Himself”, Jemmin Inc., 1983).
Sadness	164	A boxer dying after a fight and a young boy grieving. The boy cries and asks everyone to wake up the boxer. (“The Champ”, MGM, 1979)
Anger	234	A group of boys beats and humiliates another boy in front of his friends. (“My Bodyguard”, Twentieth Century Fox Film Corporation, 1980)
Fear	82	A little boy playing when a ball mysteriously trundles towards him, then he starts looking for his mother. (“The Shining”, Warner Bros, 1980)
Surprise	44	A man staying in his house. Suddenly, the door opens, and many men swoop into the house (“Capricorn One”, Associated General Films, 1977)
Disgust	65	Amputation of a hand in medical surgery. (Non-commercial)
**Arabic Clips**		
Amusement	174	A girl struggling to speak English on behalf of her mother. (“Bye-Bye London”, Funoon Play, 1981)
Sadness	59	A girl has a bad dream about her dead father and cries for missing him. (“Bu kreem with seven woman”, MBC, 2011)
Anger	133	A man beats and humiliates another man and offends his beliefs. (“Omar”, MBC and Qatar TV, 2012)
Fear	50	Two men attacking an old lady, stealing her money and injuring her hand with a knife. (“Mother is a Basha”, ART, 2013)
Surprise	45	A doctor leaving his clinic in peace. Suddenly, he gets shot by another man. (“Tagreebah Falestineeah”, Syrian Art Production International, 2004)
Disgust	30	A man eats insects. (“Arab Got Talent”, MBC, 2013)

**Table 2:** List of questions in the online survey.

Page	Questions
1	Aim, explanations and consent
2	**General:** Gender, age, living region of Saudi Arabia
	**Health:** Heart, breathing, sight and hearing issues
	Watching media habits for both English and Arabic content
	Current mood (self-assessment)
3–14	Play each clip (for 6 emotions for English and Arabic)
	**Feeling:** Rate the level of each emotion felt (0 not felt, 10 felt strongly)
	**About the clip:** - Seen before - Looked away *
	**Acceptance:** - General acceptance - Reasons for non-acceptance (if any)
15	Current mood
	**Accompany:** - Being in the company of with others while watching? - If yes, did their presence affect your answers?

* Avert one’s gaze while watching a clip to avoid feeling intense emotion (e.g., disgust).

**Table 3:** Overview of devices used for measuring physiological and behavioural responses.

Device	Extracted and Analysed Features
Microsoft Kinect	Facial Points
	Head Pose
	Facial Action Units (AUs)
Tobii Eye Tracker	Eye Movement and Activities
	Gaze
Q-sensor	Skin Conductance
	Temperature
EPOC Emotiv	Brain wave Analysis

**Table 4:** Number of responses for each clip in the online survey.

	Amusement	Sadness	Anger	Fear	Surprise	Disgust
English	345	258	226	196	182	171
Arabic	289	242	208	190	175	166

**Table 5:** Number of segments for each emotion and each device in the physiological and behavioural measurement.

	Amus.	Sad.	Anger	Fear	Surprise	Disgust
English Clips (with Arabic subtitles)						
Kinect	14	21	13	13	12	15
Tobii	17	26	19	21	22	25
Q-Sensor	16	23	19	19	19	22
Arabic Clips						
Kinect	23	14	12	11	10	15
Tobii	27	18	19	20	19	25
Q-Sensor	24	16	17	19	17	22

**Table 6:** Main reasons for rejecting the selected clips including percentage of the given reason for rejection.

Emotion	English Clips	Arabic Clips
Amusement	63% wording (curses)	56% disrespectful of parents
	55% disrespectful of religious figure	
	42% disrespectful of parents	
Sadness	-	64% loud music
Anger	59% violence (bullying)	64% depicting religious figures
Fear	-	54% assaulting a women
Surprise	-	-
Disgust	77% intensity of disgust	45% intensity of disgust

**Table 7:** Summery of selected measurements with significant differences between the 6 elicited emotions from both English and Arabic clips based on the ANOVA group test. SCL, Skin Conductivity Level.

Measurement	#	Significant Features
Facial AUs	36	Range and maximum duration of:
		- decrease and increase of All 6 AUs
		- increase velocity of AU1
		Range and minimum duration of:
		- decrease velocity of AU2
		- decrease velocity of AU3
		Rate of steady AU0 state
		Range duration of:
		- increase velocity of AU2 and AU5
		- decrease velocity of AU5
		Minimum duration of:
		- decrease velocity of AU1
		- increase velocity of AU4
		Maximum duration of:
		- increase velocity of AU5
		- continuous movement of AU3
Head Pose	17	Rate and average duration of:
		- fast yaw, roll and pitch movements
		- continuous yaw, roll and pitch movements
		Maximum duration of fast roll movement
		Range and maximum duration of anticlockwise roll
		Average and maximum duration of left yaw
Eye Activity	12	Range and maximum duration of
		- open left, right and both eyes
		Maximum duration of
		- small and large pupil size for left and right eyes
SCL	11	Duration rate of increased changes of SCL
		Maximum duration of high SCL
		Range and minimum duration of low SCL
		Average count of the occurrence of:
		- increase changes of SCL
		- steady state and continuous change of SCL
Temperature	22	Range and std of temperature
		Maximum duration of high temperature
		Average, std and var of temperature velocity changes
		std and var of temperature acceleration changes
		Average, range, minimum and maximum duration of
		low temperature
		Rate of steady state and continuous change of temperature
		Rate of increase change of temperature
		Average duration of decrease change of temperature
		Average count of occurrence of:
		- increase change of temperature
		- steady state and continuous change of temperature
Total Features	98	

#: number of total selected features based on the ANOVA group test, std: standard deviation, var: variance, For AUs’ illustration, see Figure 3.

**Table 8:** Classification accuracy (in %) for the 6 elicited emotions from English and Arabic clips.

Features	English Clips	Arabic Clips
Facial AUs	37.5	49.4
Head Pose	48.9	61.2
Eye Activity	33.8	36.7
SCL and Temperature	89.0	92.2
Average	52.3	59.9
